# Production, characterization and therapeutic efficacy of egg yolk antibodies specific to *Nosema ceranae*

**DOI:** 10.1371/journal.pone.0297864

**Published:** 2024-02-09

**Authors:** Mehmet Nuri Açık, Burcu Karagülle, Seda Yakut, Yasin Öztürk, Mehmet Ali Kutlu, Recep Kalın, Burhan Çetinkaya

**Affiliations:** 1 Department of Microbiology, Faculty of Veterinary Medicine, University of Bingol, Bingol, Turkiye; 2 Department of Microbiology, Faculty of Veterinary Medicine, University of Firat, Elazig, Turkiye; 3 Department of Histology and Embryology, Faculty of Veterinary Medicine, University of Bingol, Bingol, Turkiye; 4 Department of Pharmacology, Faculty of Veterinary Medicine, University of Necmettin Erbakan, Konya, Turkiye; 5 Department of Plant and Animal Production, Vocational School of Food, Agriculture and Livestock, University of Bingol, Bingol, Turkiye; 6 Department of Microbiology, Faculty of Veterinary Medicine, Cumhuriyet University, Sivas, Turkiye; University of British Columbia, CANADA

## Abstract

*Nosema* disease, caused by *Nosema ceranae*, one of the single-celled fungal microsporidian parasites, is one of the most important and common diseases of adult honey bees. Since fumagillin, which has been used for decades in the control of *Nosema* disease in honey bees (*Apis mellifera*), poses a toxic threat and its efficacy against *N*. *ceranae* is uncertain, there is an urgent need to develop alternative prophylactic and curative strategies for the treatment of this disease. The main aim of this study was to investigate the therapeutic potential of specific egg yolk immunoglobulins (IgY) on *Nosema* disease. For this purpose, the presence of *N*. *ceranae* was determined by microscopic and PCR methods in honey bees collected from *Nosema* suspicious colonies by conducting a field survey. Layered Ataks chickens, divided into four groups each containing 20 animals, were vaccinated with live and inactivated vaccines prepared from field isolates of *N*. *ceranae*. Eggs were collected weekly for 10 weeks following the last vaccination. IgY extraction was performed using the PEG precipitation method from egg yolks collected from each group, and the purity of the antibodies was determined by SDS-PAGE and Western Blot. The presence of *N*. *ceranae*-specific IgYs was investigated by Western Blot and indirect ELISA methods. It was determined that specific IgYs showed high therapeutic efficacy on *Nosema* disease in naturally infected bee colonies. In addition, honey bees collected from infected colonies were brought to the laboratory and placed in cages with 30 bees each, and the effectiveness of IgYs was investigated under controlled conditions. It was detected that specific IgY reduced the *Nosema* spore load and the number of infected bees significantly in both the field and experimental study groups treated for seven days. It was concluded that chicken IgYs, an innovative and eco-friendly method, had a significant potential for use as an alternative to antifungal drugs.

## Introduction

Honey bees (*Apis mellifera*) are recognized as a vital part of the global ecosystem and the world’s food supply due to their significant role in pollinating both natural and agricultural flora. In addition, many products obtained from honey bees, particularly honey, pollen, propolis, royal jelly and bee venom are widely used in public health, food and pharmaceutical industries. In recent years, there has been a serious decline in the honey bee populations all over the world owing to the destruction of habitats, intensive use of pesticides, global climate and diseases [1]. One of the most common infectious diseases affecting honey bees today is *Nosema* disease, which is caused by two different species of unicellular fungal microsporidian parasites, *Nosema apis* and *N*. *ceranae* [2, 3]. Although both species cause infection in honey bees, *N*. *ceranae* has recently been reported as the most common honey bee pathogen all over the world. *N*. *ceranae* has been associated with decreased honey production, weakness and increased mortality in asymptomatic colonies [4]. In addition, it can cause disruption of carbohydrate and lipid metabolism by disturbing intestinal absorption, change in foraging behavior, loss of motility, shortening of the life span of honey bees, immunosuppression and colony collapse in the later stages of the disease [4, 5].

Fumagillin, isolated from *Aspergillus fumigatus*, is the only approved chemical agent widely used in the treatment of *Nosema* disease [6]. Although fumagillin was first used for *N*. *apis* infections with sufficient effectiveness, it has been reported to have limited effect against *N*. *ceranae* that emerged later and spread all over the world [7, 8]. Fumagillin has been reported to reduce spore load temporarily and did not cause a significant change in colony size [8]. Long-term use of fumagillin in apiculture leads to the development of resistance and it also exacerbates *N*. *ceranae* infection at low concentrations. Fumagillin shows its effect only during the reproduction period as it interrupts the intracellular replication of *Nosema* species. However, resistant forms called spores are not affected by this drug and this poses a serious threat for honey bee health [9]. Also, fumagillin might threaten human health if its residues are found in honey [10, 11].

Fumagillin is not available everywhere: antibiotics are banned in the EU for beekeeping. So, alternative treatments are important also for situations in which fumagillin may not be available. Alternative treatment methods such as probiotics and plant extracts have been reported to be successful in controlling *Nosema* disease [12, 13]. However, the chemical structure of natural products and extracts is complex and there is a paucity of information about their content. Moreover, the production of these extracts needs to be highly standardized in order to be used for treatment in the field. For these reasons, there is an urgent need to develop alternative and innovative treatment methods with no side effects for honey bees and no residue in honey in order to prevent *Nosema* disease. In recent years, many studies have been carried out on the use of egg yolk antibodies (IgY) obtained from chickens for the treatment and control of outbreaks in bees. IgY antibodies are the predominant immunoglobulins in laying hens and transferred from serum to the yolk to provide passive immunity. Unlike antibiotics, IgY antibodies are eco-friendly and do not cause undesired side effects, resistance or toxic residues [14–16]. Successful results have been reported in the studies investigating the usability of IgY technology in the treatment and prevention of many infectious diseases in both humans and animals [17, 18]. Since antibodies are produced against different antigens of microorganisms, the risk of developing resistance is very low due to the use of polyclonal IgY against diseases [19]. Although there are a limited number of studies investigating the efficacy of IgY against honey bee diseases, no literature data are available about the use of IgY in difficult to treat honey bee diseases such as *Nosema*, hitherto. This study was therefore conducted to produce and characterize *N*. *ceranae* specific IgY from egg yolks of chickens vaccinated with vaccines prepared by four different methods. In addition, the therapeutic efficacy of the purified specific IgY was investigated in honey bees naturally infected with *N*. *ceranae*.

## Materials and methods

### Isolation and molecular identification of *N*. *ceranae*

Apiaries (n = 20) in and around Bingol province located in eastern Turkiye were visited between March and May 2022, and the colonies were examined for *Nosema* disease. A total of 20 dead or sick honey bees were collected from each of the colonies suspected of *Nosema* diseases and brought to the laboratory at Bingol University. The midguts of the honey bees were homogenized in a mortar with Phosphate Buffered Saline (PBS), and were filtered using Whatman No.4 filter paper. *Nosema* spores in the resulting suspension were purified using Percoll (Sigma-Aldrich, St. Louis, MO) and counted by a hemocytometer (Hausser Scientific) [20]. For the identification of *Nosema* spores at the species level by PCR, the suspension was centrifuged and DNA extraction was performed using QIAamp® tissue kit (Qiagen, Germany). DNA samples were amplified by multiplex PCR using two pairs of primers specific for the 16S rRNA gene of *N*. *apis* and *N*. *ceranae* [21].

### Chickens used in vaccination

A total of 100 layered Ataks chickens, 24 weeks old and weighing 2–2.5 kg, were used for vaccination. The chickens were placed in 32x60x35 cm sized cages with grills, each containing one chicken. The animals were given water and feed *ad libitum* and provided with adequate light throughout the experimental study. The chickens were divided into five groups, each containing 20 animals, as four vaccination groups and one control group.

### Preparation of inactive *Nosema* vaccine and vaccination of chickens

Two inactivated vaccines derived from *Nosema* proteins and seed culture spores were prepared to vaccinate chickens. Cell wall proteins were extracted from *Nosema* spores using the method reported by Wu et al. [22]. Briefly, 10 mL suspension containing 1x10^6^ /mL *Nosema* spores was centrifuged at 600 g for 5 min. The supernatant was discarded and the pellet was washed twice with 10 mL PBS. The resulting pellet was suspended in 200 μl extraction buffer (1% SDS, 10% sucrose, 5% β-mercaptoethanol, 60 mmol/L Tris, 100 mmol/L EDTA, 250 μM HCl_2_) and incubated at 60°C for 30 min. Following the incubation, the suspension was centrifuged at 11600 g for 10 min at 4°C and, the amount of protein in the supernatant was measured using Bicinchoninic acid (BCA) kit (Thermo Scientific) and visualized by 12% Sodium Dodecyl Sulfate Polyacrylamide Gel Electrophoresis (SDS PAGE). The second inactivated vaccine was obtained from *Nosema* spores subjected to inactivation by heat treatment. For this purpose, *Nosema* spores (1 x 10^6^/mL) were inactivated by the application of heat at 100°C for 15 min.

The vaccine emulsion was prepared by taking 0.5 mL of the suspension (1 mg/mL) containing the purified *Nosema* spore proteins and mixing it with an equal amount of Freund’s complete adjuvant (FCA). The chickens were vaccinated with 1 mL of inactivated vaccine at three different sites (front, right and left side of the pectoral muscle) by intramuscular administration (Group 1). Following the first vaccination, 0.5 mL of the vaccines prepared with Freund’s incomplete adjuvant (FIA) were administered twice to chickens intramuscularly at two week intervals. The seed culture vaccines prepared by inactivating *Nosema* spores with heat were also given to the chickens as stated above (Group 2). Sterile physiological saline was administered to the unvaccinated chickens with the same method (Control Group). Following the last inoculation, the eggs were collected weekly for 10 weeks and stored at 4°C until use.

### Preparation of live *Nosema* vaccine and vaccination of chickens

Studies have showed that *Nosema* spores lose their viability and infectivity significantly during freezing and thawing [23, 24]. Therefore, fresh unfrozen *Nosema* spores isolated in the field were used to vaccinate chickens. Two different methods were used to inoculate chickens with live vaccines. In the first method, 0.5 mL of the suspension containing *Nosema* spores (10^6^/mL) was mixed with an equal amount of FCA to prepare the vaccine emulsion. The vaccine containing 1 mL of live *Nosema* spores was inoculated by intramuscular administration to chickens at three different sites (front, right and left side of the pectoral muscle). After the first vaccination, 0.5 mL of the vaccines prepared with FIA were applied twice to chickens intramuscularly at two week intervals (Group 3). In the second method, 20 mL solution containing 10^6^/mL live *Nosema* spores was added to the drinking water of chickens for oral vaccination. The chickens were revaccinated in the same way two weeks after the first vaccination (Group 4). Following the last inoculation, the eggs were collected weekly and stored at 4°C until use.

### Purification of IgY antibodies from egg yolk

Eggs collected weekly after the last vaccination were disinfected with 75% alcohol and then the yolk was separated from the albumen. The yolk was transferred to a filter paper and rolled to remove the remaining albumen, and then the yolk was cut with a lancet and transferred to 50 mL falcon tubes. IgY was purified from the egg yolks by using 3.5%, 8.5% and 12% PEG6000, respectively [25, 26]. IgYs produced from egg yolk of chickens in each group were pooled to obtain multiple polyclonal antibodies. The purified IgY antibody concentration was measured by using BCA kit and sterilized with a 0.22 μm membrane filter. IgY antibodies were transferred to Eppendorf tubes and stored at -20°C for later analysis. The purity of IgY was determined by SDS-PAGE and Western Blot [14, 27].

### Characterization of *N*. *ceranae* specific IgYs by ELISA and Western Blot

ELISA polystyrene microplates were coated with PBS containing *N*. *ceranae* antigen at a concentration of 1 μg/mL and incubated overnight at 4°C. Then, the wells were blocked with the blocking solution (PBS with 0.5% Bovine Serum Albumin, pH 7.4, 200 μL /well) and incubated at room temperature for 2 hours. The blocking solution was removed using the washing solution (PBS with 0.05 Tween-20, 250 μL /well). A pool of purified antibody samples from each group that was generated from chickens laying every week during a ten-week period was diluted in 1/100 and added to the wells (100 μL / well), and then incubated at room temperature for 2 hours. Afterwards, the wells were washed three times with the washing solution and goat anti-chicken IgY antibody (100 uL / well) conjugated with horseradish peroxidase (ab97135, Abcam-UK) was added to each well and incubated at room temperature for 1 hour. The plates were then washed 5 times with the washing solution and substrate solution (TMB-tetramethylbenzidine dihydrochloride, 100 μL /well) was added to the wells. After incubating the plates in the dark for 10 min, the reaction was stopped by adding 1 M H_2_S0_4_ (50 μL/well). Optical density was measured at 450 nm in the ELISA reader [28]. Maximum dilution of sample was determined as the IgY titer, while the OD_sample_/OD_negative_ was ≥2.1.

*Nosema* proteins purified from spores were analyzed by SDS-PAGE electrophoresis to detect *N*. *ceranae* specific IgYs obtained from egg yolks. Following electrophoresis, the gels were separated from the glass plates and Western Blot analysis was carried out. For this purpose, blotting was performed to transfer protein bands to the nitrocellulose membrane. After blotting, the nitrocellulose membranes removed from the cassette were washed with Tris buffer solution (TBS-T, 0.02 M, pH 7.6) containing 0.05% Tween 20. In the second step, unrelated protein binding sites on the nitrocellulose membrane were blocked in order to prevent nonspecific reactions. Following blocking, primary antibodies containing *Nosema* specific antibodies obtained from chickens with 1:100 dilution were incubated for 2 hours. The nitrocellulose membranes were incubated with conjugated rabbit anti-chicken IgY (A9046, Sigma-Aldrich, Saint-Louis, MO, USA) diluted in 1:5000 after washing 5 times with TBS-T at 5 min intervals. The washing procedure was repeated under the same conditions and in the final step, protein bands were visualized using 3,3′-Diaminobenzidine (DAB) [14].

### Efficacy of specific IgY in the treatment of *Nosema* disease

#### Cage trials

One of the apiaries that was diagnosed to be positive for *N*. *ceranae* by microscopic and PCR examination was revisited between May and June 2023 to collect infected honey bees for cage trials. For this purpose, 60 honey bees randomly collected from the frames at the entrance of three infected colonies (20 from each) were placed in air-permeable jars and transported instantly to the laboratory. The bees in positive control were randomly placed in 20x16 cm cages with wire on three sides and wood on the other side, each containing 30 honey bees. In addition, as negative control, a total of 30 honey bees randomly collected from three colonies (10 from each) that were negative for *Nosema* disease from a different apiary located in a different region were brought to the laboratory under the aforementioned conditions and placed in a separate cage. The cage trials were performed in three repetitions at different time periods. In order to avoid cross contamination, the honey bees in each group were kept in independent cages and in different incubators at 34 ± 2°C and 60% humidity throughout the experiment [29].

Honey bees were fed with 50% sucrose solution *ad libitum* during the experiment. In addition, a commercial mixture of amino acids and vitamins (BEELOVE FLORA, Ekin premix, Mersin, Turkiye) was given to the bees as a dietary supplement. The honey bees were not supplemented with honey and pollen owing to the possibility of carrying *Nosema* spores. Because the potential of polyclonal antibody diversity in egg yolks of chickens vaccinated with whole *Nosema* spores was higher, treatment trials were carried out with IgYs purified from chicken eggs in the weeks when the highest titer was obtained following the intramuscular inoculation of live *Nosema* spores. 30 mL of 50% sucrose solution containing purified specific IgY (50μg/mL) was given daily fresh to the treatment group one day after the honey bees were placed in the cages. The other two groups (one positive and one negative control group) were not treated during the experiment. During the seven day experiment period, the honey bees were checked daily and the dead ones were removed from the cages and stored for later use. At the end of the study, 10 honey bees euthanized on dry ice were examined in terms of the number of infected bees and spore load.

In order to determine the spore load, the midguts of 10 honey bees per group were individually homogenized in 1 ml of sterile PBS and the *Nosema* spore numbers per bee were counted using a hemocytometer. The spore load per bee was calculated according to the formula: Z = α/β × δ × 250,000 (Z = spore number per bee, α = total number of spores counted, β = number of squares counted, δ = dilution factor) [30].

In order to determine the number of infected bees, the midguts of each honey bee were placed in separate Eppendorf tubes and crushed in PBS, and examined microscopically for *Nosema* spores. Honey bees with detectable *Nosema* spores were considered infected.

The midguts of four honey bees from each group were removed and fixed in 10% buffered formaldehyde solution for histopathological examination. Following 48 hours of fixation, the samples were transferred to an auto technic devise (TP 1020, Leica, Germany) containing alcohol, xylene and paraffin series at varying degrees (70, 80, 90, 100%). The honey bees blocked with granulated paraffin were cut with a microtome (RM 2155, Leica, Germany) at a thickness of 5 μm. The tissues were stained with Hematoxylin-Eosin to evaluate the presence and severity of inflammatory reaction. The stained tissues were examined under a light microscope (DM 2500, Leica, Germany) [31].

#### Field trials

The field trial was carried out in an apiary with 20 hives, which consented to the study, to determine the effectiveness of specific IgY on infected honey bees under natural conditions and then to compare the results with those of cage trials performed under controlled conditions The anamnesis about the disease was obtained from the owner before the treatment protocol was applied. The field trials were carried out in an apiary (38°53′05.9″N 40°30′21.9″E) located in the countryside of Bingol province in June 2023. Three *Nosema*-positive colonies in this apiary were used for the field trial. These colonies were moved to a separate place, approximately 500 m far away from the other colonies in the apiary before starting the treatment procedure. A total of 30 honey bees (15 for spore load and 15 for determining the number of infected honey bees) were randomly taken from the frames at the entrance of each colony prior to administrating specific IgY to the honey bees. Sample collection from adult bees was carried out in the afternoon. Maximum attention was paid not to take samples from young bees, which are less likely to carry *Nosema*. Adult bees collected by hand were placed in ventilated jars and transported to the laboratory in a short time. Honey bees euthanized on dry ice were examined in terms of the number of infected individuals and spore load as described above.

50 mL of 50% sucrose solution containing purified specific IgY (50μg/mL) were given fresh daily per colony. It was observed that all of the given solution was taken up by the bees. The apiaries were visited daily for seven days and the treatment was repeated. The number of dead honey bees were recorded using dead bee traps. The health status of the honey bee colonies was checked in the colonies at each visit by one of the authors (M. Ali Kutlu) who is a licensed specialist in beekeeping. The health status of the bees were evaluated by considering parameters such as the presence and performance of the queen bee, colony dynamics, production in the colony, physiological characteristics (movement, flight), ectoparasites, specific clinical signs indicating infectious diseases [32]. At the end of treatment period, 30 honey bees randomly collected from each colony were used for determining the number of infected honey bees and spore load as described above.

### Statistical analysis

In this study, descriptive statistical data were presented as percentage for categorical variables, and as mean ± standard deviation for continuous variables. The Shapiro-Wilk test was used to determine the normality of data distributions, while the homogeneity of variance of the groups was investigated by the Levene test. One-way analysis of variance (One-way ANOVA) was applied for the data compensating parametric assumptions, and post hoc Tukey test was used to determine differences between the groups. The comparison of two independent and normally distributed continuous variables was carried out with the independent sample T test, whereas the comparison of two continuous variables that were dependent and not normally distributed was made with the Wilcoxon Signed Rank test. A chi-squared test was used to examine the relationship between independent categorical variables, while Mc Nemar test was employed to determine the relationship between dependent categorical variables. SPSS (IBM SPSS Statistics Desktop 20.0) program was used to perform the statistical tests.

### Ethics statement

Ethical approval for the chicken study was obtained from Bingol University Animal Experiments Local Ethics Commission, Turkiye (29/06/2021–03–02). According to the regulation on the working procedures and principles of the Animal Experiments Ethics Committees of the Ministry of Agriculture and Forestry, no ethical approval was required for studies performed on bees. The honey bees used for this study were euthanized humanely by placement on dry ice. All honey bees were collected with permission from the apiary owners. In addition, the field trial was carried out in an apiary with 20 colonies, which consented to the study. Apiary owners were informed about the study and their consent documents were signed and recorded.

## Results

### Isolation and molecular identification of *N*. *ceranae*

In the microscopic examination, three (15%) of 20 apiaries were found to be positive for *Nosema* spp. in the field study. As a result of multiplex PCR analysis for species determination, band profiles specific to *N*. *cerenae* at the size of 218 bp were detected in the samples collected from these three apiaries.

### Vaccination of chickens

In the examination of the chicken group vaccinated by intramuscular injection, no complications or side effects, especially an inflammatory reaction at the vaccination site, were determined. All the animals appeared healthy during and after the vaccination. No pain, discomfort or tissue damage was detected in the animals during the palpation of the inoculation sites. The procedures and manipulations were shortened (three min per chicken) in order to keep stress levels of the chickens at minimum. Despite this, it was observed that there was no homogeneity in the daily egg production of the chickens following vaccination. Although no decrease was detected in the egg laying capacity, some chickens did not lay periodically during the time periods when the eggs were collected. For this reason, IgY trends of eggs belonging to 10 chickens in each group which laid periodically during the 10 week period after vaccination were evaluated.

### Protein purification from *N*. *ceranae*

**Fig 1:**
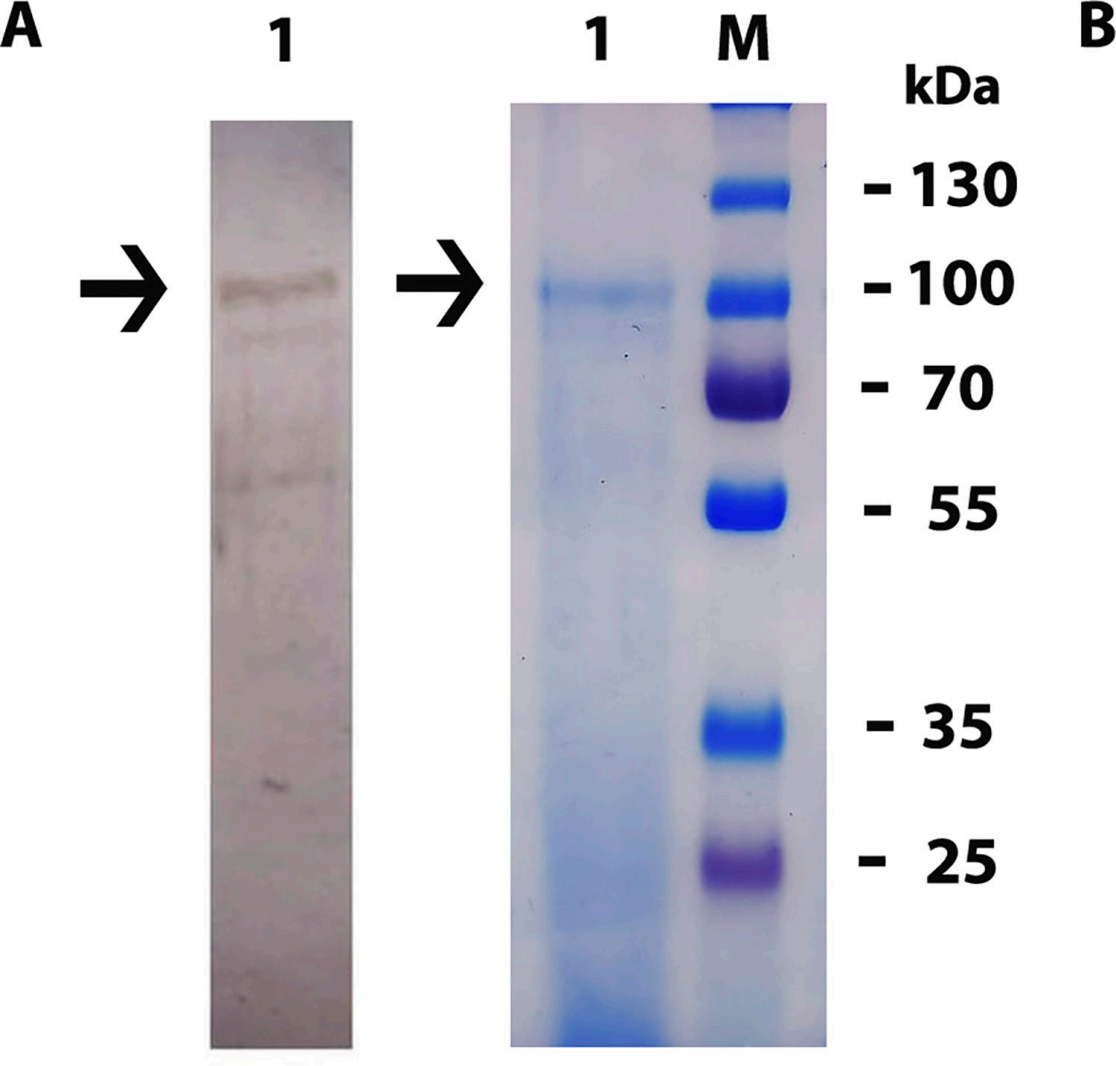
Characteristics of the purified proteins and IgY antibodies specific to *N*. *ceranae*. (A) 1: Western blot analysis of the specific IgY antibody (B) 1: SDS-PAGE analysis of the purified proteins, M: Pre-stained Protein Ladder (Thermo Fisher Scientific).

The concentration of *Nosema* spore protein was determined as 1.84 mg/mL by the extraction method with SDS lysis buffer. SDS-PAGE analysis of *N*. *ceranae* surface proteins produced one main band at the size of approximately 100 kDa (Fig 1B).

### Purification and characterization of IgY

**Fig 2:**
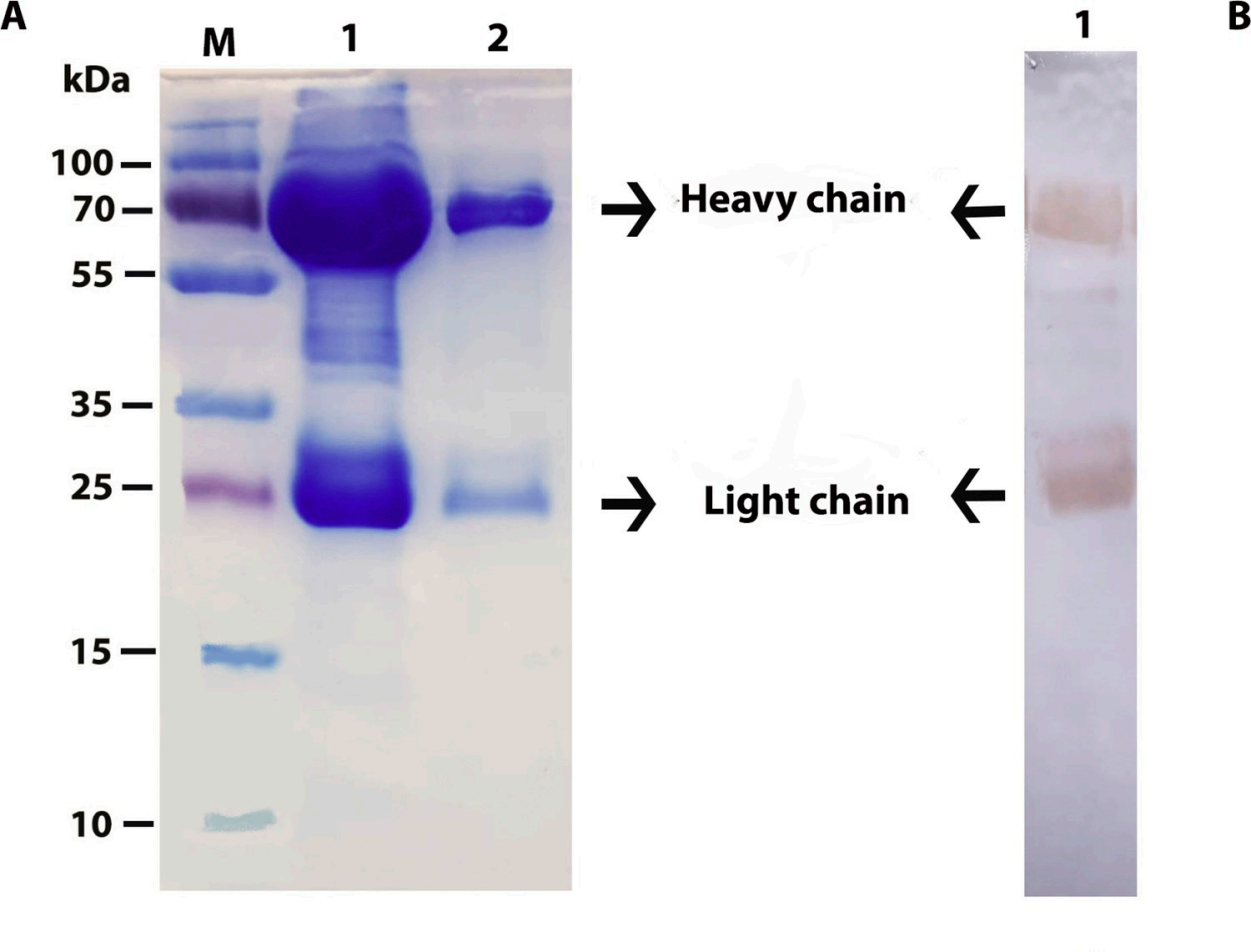
SDS-PAGE and Western Blot profiles of total IgY in egg yolk. (A) 1–2: SDS-PAGE analysis of the purified IgY and 1/10 diluted IgY, (B) 1: Western Blot analysis of IgY, M: Pre-stained Protein Ladder.

In the SDS-PAGE gel electrophoresis of IgY antibodies purified by PEG precipitation method, two protein bands at the molecular sizes of 68 kDa and 27 kDa were observed (Fig 2A). Also, Western Blot analysis produced specific bands containing heavy and light chain proteins that verified the results of SDS-PAGE (Fig 2B).

### Demonstration of *N*. *ceranae* specific antibodies by Western Blot

Western Blot analysis of the proteins extracted from *N*. *ceranae* following SDS-PAGE analysis showed that antibodies specific to the agent were present in the purified total IgY and they could bind with the proteins (Fig 1A).

### Detection of *N*. *ceranae* specific antibodies by ELISA

**Fig 3:**
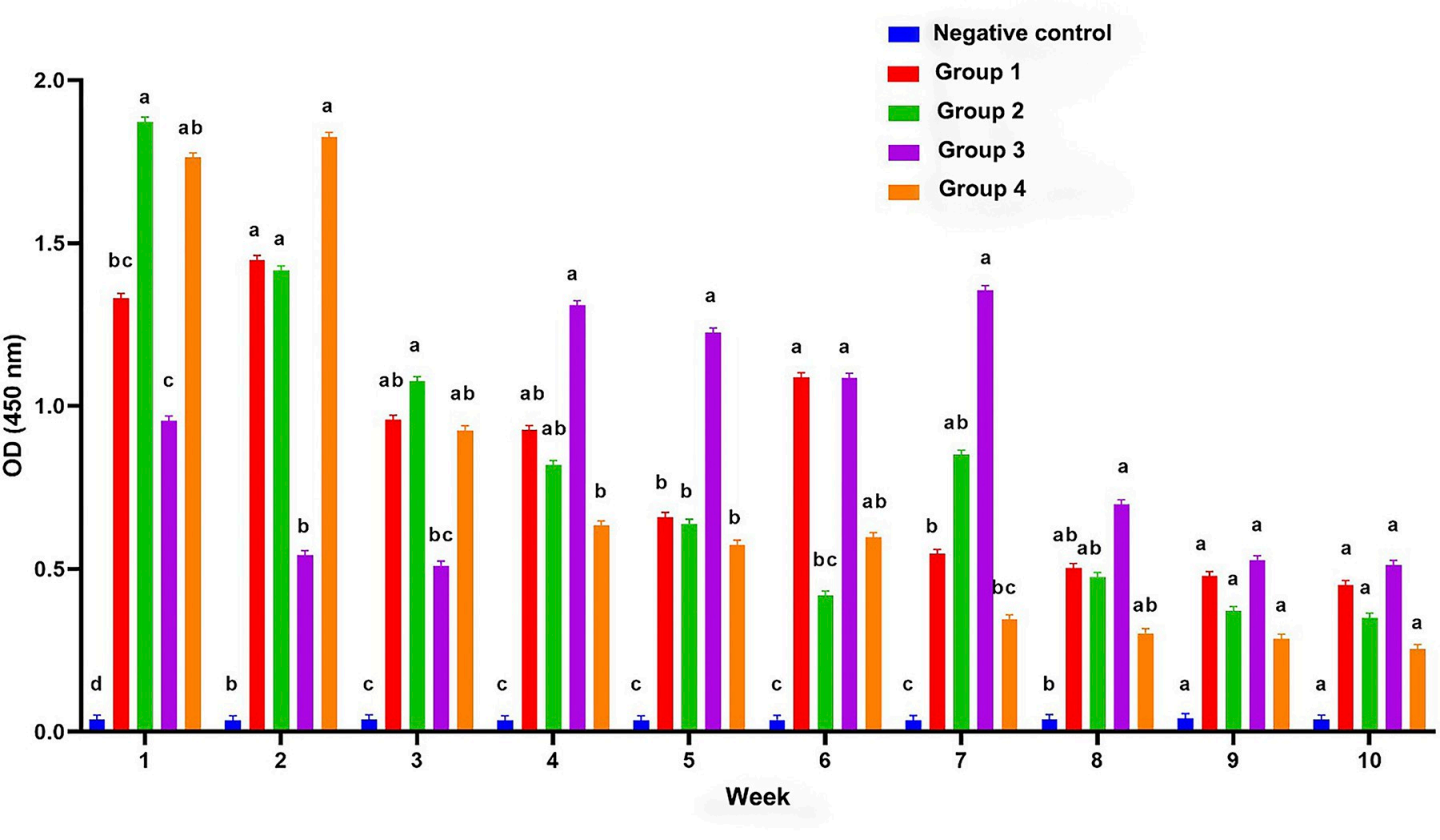
Weekly distribution of IgY titers specific to *N*. *ceranae* spores in the chicken groups. The data were presented as mean ± standard deviation^a-d^. The difference between the groups within the same week, expressed with different letter, was statistically significant (p<0.05). One-way ANOVA was used in the comparison of the groups and Post Hoc Tukey test was employed in the detection of the groups causing the difference.

An indirect ELISA was carried out to measure the immune response of purified IgY. It was observed that the antibody titer in orally vaccinated animals was rather high in the first two weeks, but decreased dramatically in the following weeks. In animals vaccinated intramuscularly with live *Nosema* seed culture vaccine, it was determined that the antibody titer was low at the beginning, remained high between the 4^th^ and 7^th^ weeks, but decreased again afterwards. In general, the antibody titer was detected to decrease dramatically from 9^th^ week, but the difference between the vaccinated and control group was not statistically significant (p>0.05) (Fig 3).

### Therapeutic efficacy of specific IgY on *Nosema* disease

#### Findings of cage trials

In three repetitions, the proportion of infected honey bees in the group treated with specific IgY ranged from 0% to 10%, whereas it was found to be between 60% and 70% in the positive control group. It was also determined that *Nosema* spore load in the treatment group decreased by 88–93% when compared to the positive control group. The differences in *Nosema* spore load and the proportion of infected honey bees between the treatment and positive control group were found to be statistically significant (p<0.05) (Table 1). The spore load was determined to be between 2.22 x 10^6^ and 2.43 x 10^6^ in the positive control group, whereas it was found to vary from 2.05 x 10^5^ to 2.18 x 10^5^ in the treatment group. *Nosema* spores were not observed in the microscopic examination of the bees in the negative control group. On the other hand, when the number of dead honey bees in the IgY-treated group was compared with the positive and negative control groups, no significant difference was detected (p>0.05) (Table 2). It was determined that the number of bees survived in the treatment group was 90% or more in all three replicates. Although the number of surviving bees was higher in the treatment group compared to the positive control group, this difference was not found to be statistically significant (p>0.05).

**Table 1:** Distribution of the number of infected honey bees and spore load according to the groups in the cage trials.

Repetitions	Groups	Number of infected bees (n = 10)	Number of non- infected bees (n = 10)	*X* ^2^	p^a^	Spore load (Mean± standard deviation) (n = 10)	p
1^st^ repetition	Negative control	0 (0%)	10 (100%)	5.495	0.057	0 (0%)	**0.001** *
	Positive control	6 (60%)	4 (40%)			2.43 x 10^6^ ± 0.01x10^6^	
	Treatment group	1 (10%)	9 (90%)			2.14x 10^5^ ± 0.01x 10^5^	
2^nd^ repetition	Negative control	0 (0%)	10 (100%)	8.571	0.011*	0 (0%)	**0.002** *
	Positive control	6 (60%)	4 (40%)			2.22 x 10^6^± 0.14 x 10^6^	
	Treatment group	0 (0%)	10 (100%)			2.05 x 10^5^± 0.14 x 10^5^	
3^rd^ repetition	Negative control	0 (0%)	10 (100%)	7.500	**0.020** *	0 (%0)	**0.002** *
	Positive control	7 (70%)	3 (30%)			2.35 x 10^6^ ± 0.14 x 10^6^	
	Treatment group	1 (10%)	9 (90%)			2.18 x 10^5^± 0.14 x 10^5^	

Data were presented as mean ± standard deviation for spore load, and as percentage for infected and non-infected honey bee numbers. X^2^: chi squared, ^a^Pearson chi squared.

*: Difference between the groups was compared with Independent Samples T test (p<0.05).

**Table 2:** Distribution of the dead and survived honey bees according to the groups in the cage trials.

Repetitions	Groups	Number of bees that died during the trial (n = 30)	Number of bees that survived at the end of the trial (n = 30)	*X* ^2^	p^a^
1^st^ repetition	Negative control	2 (6.7%)	28 (93.3%)	2.693	0.260*
	Positive control	6 (20%)	24 (80%)		
	Treatment group	3 (10%)	27 (90%)		
2^nd^ repetition	Negative control	2 (6.7%)	28 (93.3%)	1.098	0.578*
	Positive control	4 (13.3%)	26 (86.7%)		
	Treatment group	2 (6.7%)	28 (93.3%)		
3^rd^ repetition	Negative control	1 (3.33%)	29 (96.7%)	1.921	0.383*
	Positive control	4 (13.3%)	26 (86.7%)		
	Treatment group	3 (10%)	27 (90%)		

Data were presented as percentage for the number of dead/live honey bees. X^2^: chi squared, ^a^Pearson chi squared.

*: Difference between the groups was compared with Independent Samples T test (p>0.05).

### Histopathological findings

In the macroscopic examination, it was observed that the honey bees in the negative control group preserved the thickness of the midgut wall and the midgut content was in natural brown tones. The thickness of the midgut wall and color of its content in the treatment group was determined to be similar to the negative control group. However, the midgut wall of the honey bees in the positive control group was observed to become thinner (membrane-like, transparent appearance) and the color of the midgut content was paler and more fluid.

**Fig 4:**
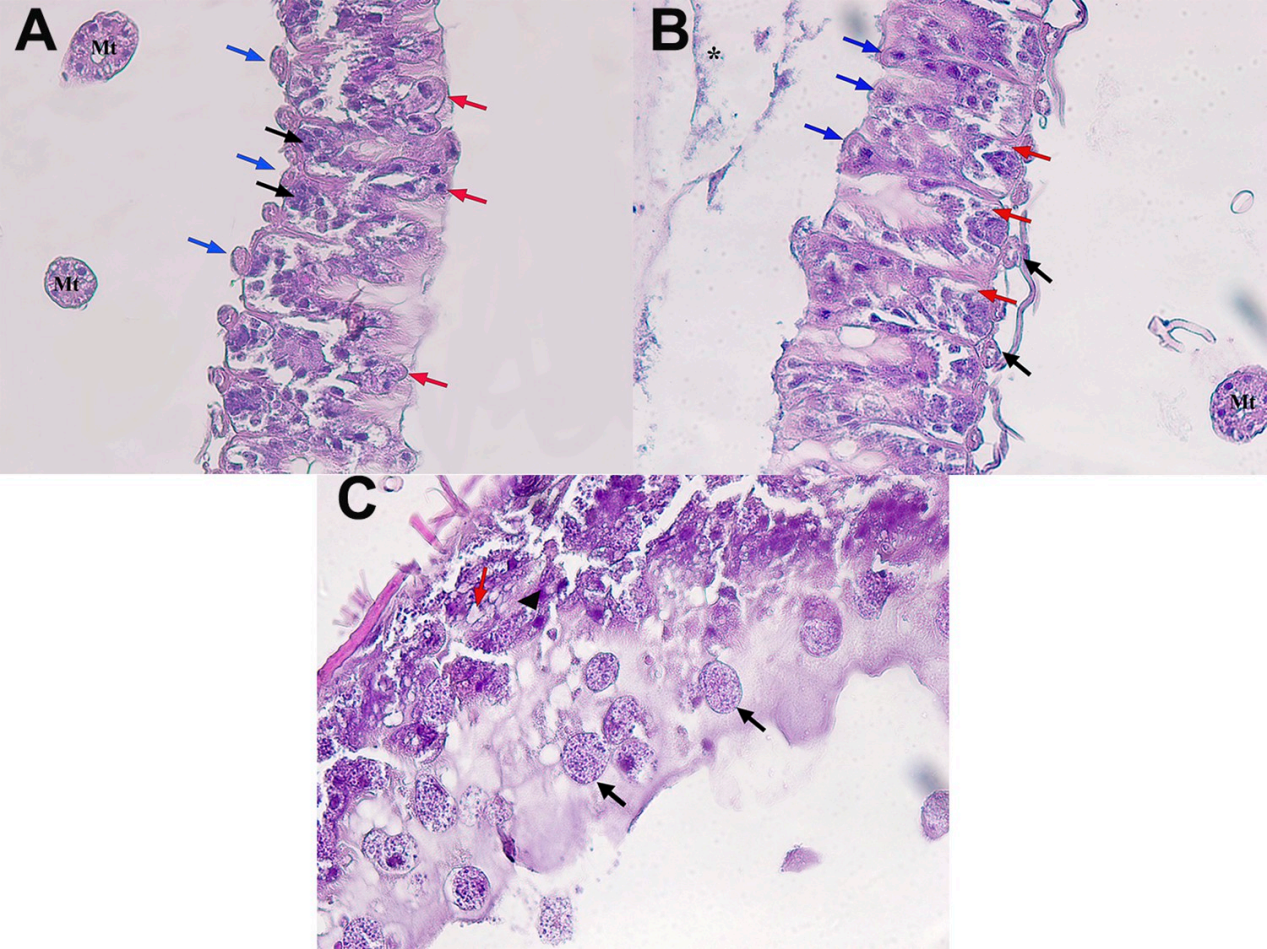
Histopathology of honey bee midguts 7 days post-treatment. Light microscopic image of honey bee midguts in the control (A), positive (B) and treatment groups (C). HxE staining, x40 magnification. A: Columnar epithelial cells (black arrow), basal cells (red arrow), muscle layer (blue arrow). Malpighi tubes (Mt). B: The epithelial cells were heavily infected with *N*. *ceranae*. Cytoplasmic droplets filled with spores protruding towards the lumen (black arrow), severe vacuolization in some epithelial cells (red arrow), a pyknotic, apically displaced nucleus (arrowhead) in epithelial cells. C: Columnar epithelial cells (blue arrow), basal cells with mild vacuolization (red arrow), muscle layer (black arrow), peritrophic membrane (asterisk) and limited regenerative areas inside epithelial folds. Malpighi tubes (Mt).

In the microscopic examination, it was seen that gastric epithelial cells and cell membranes were prominent and preserved their integrity in the negative control group. While cell nuclei appeared normal, the cytoplasms had a homogeneous and dense structure. No destruction was observed in epithelial cells, and peritrophic membrane was quite thick, dense and evenly distributed throughout the lumen (Fig 4A). In the positive control group, intense degenerative processes were detected in the gastric mucosa of the honey bees due to *Nosema* invasion. It was noted that the cell membranes were damaged or completely burst due to the increase in intracellular osmotic pressure. That’s why the borders and connections between the cells disappeared. The cell nucleus was observed to disappear in some cells. The cell cytoplasms lost their homogeneous appearance and severe vacuolization was detected. The structure of the peritrophic membrane was loosened and exhibited a diffused appearance due to volume lose (Fig 4B). Limited regenerative areas were detected in the peritrophic membrane and inside the epithelial folds in the treatment group. Mild vacuolization was observed in the epithelial cells (Fig 4C).

### Findings of field trials

The owner of the apiary that was subjected to treatment for *Nosema* stated that 40–45 honey bees died in each colony on average within 15 days following the onset of the disease. It was detected that the honey bees died or about to die at the entrance of the infected colonies, but no signs of disease were observed in the honey bees at all. Although the number of bees died at the entrance of all three colonies was high in the first days of the treatment, it was observed to decrease gradual afterwards (Table 3).

**Table 3:** Number of honey bee died during the field trials.

Colonies	1^st^ day	2^nd^ day	3^rd^ day	4^th^ day	5^th^ day	6^th^ day	7^th^ day
1^st^ colony	40	38	22	17	9	5	4
2^nd^ colony	42	34	28	15	11	7	5
3^rd^ colony	45	40	25	16	8	5	3
Average	42.3	37.3	25	16	9.3	5.7	4

At the onset of the field trial, the proportion of infected honey bees in the three colonies varied from 66.7% to 86.7%, but it decreased significantly down to 13–20% after the treatment with specific IgY. Likewise, the spore load that was between 2.73 x 10^7^ and 3.15 x 10^7^ at the beginning decreased to the values ranging from 4.15 x 10^3^ to 1,85 x 10^4^ following the treatment. The differences between spore load and the number of infected honey bees before and after the treatment were statistically significant (p<0.05) (Table 4).

**Table 4:** Number of infected honey bees and distribution of spore load before and after treatment in three colonies.

	1^st^ colony	2^nd^ colony	3^rd^ colony
	Number of infected bees (n = 15)		
Pre-treatment	12 (80%)	10 (66.7%)	13 (86.7%)
Post-treatment	2 (13.4%)	2 (13.4%)	3 (20%)
P*	**0.002**	**0.008**	**0.002**
	Spore counts (n = 15)		
Pre-treatment	2.73 x 10^7^ ± 0.1 x 10^7^	2.22 x 10^7^± 0.1 x 10^7^	3.15 x 10^7^ ± 0.1 x 10^7^
Post-treatment	1.14 x 10^4^ ± 0.1 x 10^4^	1.85 x 10^4^ x 0.1 x 10^4^	4.15 x 10^3^ ± 0.1 x 10^3^
P^a^	**<0.001**	**<0.001**	**<0.001**

The numbers of infected bees in the groups were presented as percentage.

P*: The differences between the number of infected bees in the same groups before and after treatment were determined by the Mc Nemar Test (p<0.05). The spore numbers of the groups were presented as mean ± standard deviation.

P^a^: The differences between the number of spores in the same groups before and after treatment were determined by Wilcoxon Test (p<0.05).

## Discussion

In the present study, purification of *N*. *ceranae*—specific IgYs from chicken egg yolks was successfully performed for the first time. Studies have showed that IgY antibody production is affected by many factors including vaccination protocols, adjuvant types, extraction techniques and quality and dose of antigens [16, 33]. The researchers generally prefer vaccinating chickens by intramuscular route at certain intervals for IgY production. Side effects of the adjuvants used and reduction in laying capacity are among the major problems of intramuscular vaccination. It has been reported that FCA, which is frequently used as an adjuvant, may lead to stress due to inflammatory and granulomatous lesions at the injection site which results in decrease or even complete cessation in laying capacity of chickens [34]. Stills [35] reported that possible side effects due to FCA can be minimized by using formulations with limited mycobacterial content and by delivering low volumes of the total vaccine to more than one site. As a matter of fact, the application of low-volume vaccinations at different sites of chickens using FCA has been demonstrated not to cause any side effects or decrease in laying capacity [36, 37]. In parallel with the abovementioned studies, no side effects or decreases in egg capacity were observed in chickens following the vaccination using FCA and then FIA in the current study. These findings supported that the administration of vaccines combined with FCA was a good option for the production of polyclonal antibodies against *N*. *ceranae* in chickens. However, the European Union Reference Laboratory for Alternatives to Animal Testing (EURL ECVAM) recommends that “animals should no longer be used for the development and production of antibodies for research, regulatory, diagnostic and therapeutic applications” due to possible suffering of animals [38]. For this reason, oral vaccination has been suggested as an alternative method in order to eliminate possible injection-induced side effects and to minimize the stress that can occur during holding animals [39–41]. In this study, both oral and intramuscular administration were used to determine the most effective immunization protocol, as the chickens were vaccinated for the first time with the vaccines prepared from *Nosema* spores.

There is no consensus in the literature regarding the number and intervals of vaccination in chickens, and egg collection times for the production of specific IgY. Vaccination numbers ranging from three to five have been applied in studies employing injection [28, 42, 43]. In a study conducted on *Acinetobacter baumanni*, the highest IgY titer has been achieved after three injections, but the fourth vaccination did not cause an increase in the antibody titer [44]. In previous studies, egg collection following the first vaccination has frequently been applied to observe the pattern of IgY titers in the yolk. In vaccination with this method, antibody titers have been reported to reach the peak level after the last vaccination [28, 42, 45]. For these reasons, a weekly egg collection protocol was applied after three dose application of the vaccine in order to obtain the highest antibody titer in the current study.

While the antibody titer was higher in the first two weeks following vaccination with inactivated vaccines, it was observed that it was higher between 4^th^ and 7h week of vaccination with live vaccines. IgY titers have been reported to show fluctuations over time in several previous studies [46, 47]. Although the main cause of this fluctuation has not been fully understood, it was likely due to the irregular antibody transfer from serum to yolk among chickens, because IgY extraction has been performed from pools containing large numbers of inoculated egg yolks. The breed of chickens used in vaccination may also be responsible for this. The amount of yolk IgY produced by Rhode Island Red chicken eggs has been reported to be more than twice compared to Single Comb White Leghorns eggs, which puts forward the importance of breed selection for obtaining high antibody yield [15]. In the present study, Ataks chickens were used for the first time for IgY production and it was therefore impossible to compare their productivity with other breeds. Studies on different chicken breeds are needed to determine the highest and long-lasting antibody response against *Nosema* vaccines.

It was observed that while the antibody titer was high in the first two weeks following oral vaccination, it gradually decreased over time in the following weeks, in this study. Although no data are available on *N*. *ceranae*, controversial results have been reported for oral vaccinations with other pathogens. In a study conducted by Thibodeau et al. [48], chickens vaccinated with *Campylobacter jejuni* antigens by different routes were evaluated for the production of IgY and, the results indicated that oral vaccination stimulated significantly less antibody production compared to the other routes. On the other hand, oral vaccination of quails has been reported to produce sufficient IgY specific for *Helicobacter pylori* provided that the vaccination intervals were shorter and the number of boosters were increased [49]. In the current study, IgY concentration was detected to increase in a short time and reached a peak level on the 14^th^ day following oral administration with live *N*. *ceranae* spores twice. Similarly, Najdi et al. [49] reported that antibodies post-vaccination peaked on the 28^th^ day in quails, and then decreased dramatically. Based on these findings, it is plausible to suggest that antigens inoculated orally are removed rapidly from the body of the birds and do not stimulate the immune system well. On the other hand, it has been reported that antibody titer in chickens immunized orally with Salmonella Typhimurium or *S*. Enteritidis started to decrease 56^th^ day after vaccination, and rose again on the 63^rd^ day following revaccination [50]. Oral vaccination of chickens with *N*. *ceranae* at frequent intervals may also keep the antibody level at high concentrations for a longer period of time. If this is proven in future studies, it may be possible to use oral vaccination as a reliable alternative to injection.

One of the most common chemicals used for prophylactic and therapeutic purposes against *Nosema* disease is fumagillin. However, recent studies suggested that fumagillin had negative effects on *N*. *ceranae*. In a study conducted by Huang et al. [51], different concentrations of fumagillin have been demonstrated to exacerbate *N*. *ceranae* infection, rather than suppressing, and caused serious damage to honey bee physiology. In recent years, there has been a great increase in studies on the use of IgY in bacterial, viral, fungal and parasitic infections. Promising results have been reported in experimental studies investigating the use of IgY as an alternative to antibiotics. IgY technology has also been used against some honey bee diseases in recent years. It has been showed that IgY antibodies protected honey bee larvae against Deformed Wing virus by approximately 50% [52]. The most comprehensive study on IgY activity in honey bee diseases has been conducted by Sun et al. [28] on Chinese Sacbrood virus (CSBV). The researchers reported very high recovery proportions (95–100%) as a result of the treatment with IgY in honey bees infected with this virus. In the present study, the therapeutic efficacy of specific IgY was determined both on naturally infected colonies and on the groups formed by placing naturally infected honey bees in cages. The field trails were conducted in a limited number of colonies owing to the difficulties faced in the field study. Also, the absence of positive and negative controls, and repetitions in the field trials can be considered as the main drawbacks of this study. Despite these limitations, the evaluation of the specific IgY activity in naturally infected colonies was believed to be appreciable for future studies. As a result of the seven-day field trial, the significant decreases in the number of infected honey bees and *Nosema* spore load suggested that the therapeutic efficacy of IgY was high. However, large-scaled field studies containing control groups in different apiaries are required to reveal more accurate scientific data on the efficacy of IgY.

In experimental studies on *N*. *ceranae*, artificial infection was generally executed with young bees obtained by keeping the combs in a laboratory environment [29, 53–55]. This method is suitable to ensure that all bees used in the experiment are infected. In previous studies, inoculum application was carried out after anesthesia with CO_2_ to ensure that individual bees received all *Nosema* spores [29, 56–57]. However, this method was abandoned after it was reported that CO_2_ anesthesia affected the mortality rates and lifespans of bees and caused stress due to keeping bees [58, 59]. In recent years, individual inoculation in bees has been replaced by mass inoculation [60–62]. However, it is not possible to say that all bees are 100% infected due to the possibility that some bees may not receive a sufficient dose of *N*. *ceranae* in mass inoculations in cage experiments. In the majority of artificially created infections, a few day old young bees obtained by incubating the combs in the laboratory under appropriate conditions are used [29, 53–55]. Owing to the fact that *N*. *ceranae* generally causes more severe infections in adult bees, using adult bees in experiments seems more realistic in terms of revealing the true pathogenicity of *N*. *ceranae*. We therefore believe that investigating the effect of IgY on naturally infected adult bees in this study will yield results closer to reality. Although the prevalence of *N*. *ceranae* infection was higher in older bees, it is not possible to say that all bees used in the study were infected. However, compared to the negative control group under the same conditions, the statistical differences between the spore load and the number of infected bees indicate that IgY may have therapeutic potential.

When compared to the field trials, *Nosema* spore load was detected to be higher in the group treated with IgY in the cage trials in this study. The fact that sample size was small in both trials might be responsible for this difference. The effect mechanism of IgY may have also played role in this difference. In general, IgYs inhibit the growth and colonization of bacteria by increasing the phagocytic ability of macrophages [63]. Neutralization, inhibition of adhesion and enzymatic activity are among the effect mechanisms of IgYs, as well [64]. Since honey bees do not have a well-developed immune system as in mammals, it does not seem possible for IgY to perform all these activities. It is therefore likely that the functions of IgY on honey bee pathogens are limited to neutralization and eventually inhibition of adhesion. This view was supported by Sun et al. [28] who revealed that specific IgYs neutralized CSBV at a certain titer and prolonged the lifespan of larvae. Although the activity mechanism of IgY in preventing *Nosema* infections was not investigated in this study, it is believed that specific antibodies acted by neutralizing *Nosema* spores as in other pathogens. However, bearing in mind IgYs neutralize *Nosema* spores, honey bees must defecate for reducing the spore load in the gut. Honey bees are reluctant to defecate in cages as they normally defecate outside the colony on clean-up flights [65]. This may explain why the spore load was not decreased at a desired rate in the cage trials. Indeed, worker bees housed in the laboratory have been showed to carry more bacteria in their guts compared to those in the colonies which supported this view [66].

In contrast to the field trials, it was observed that IgY had no effect on mortality rates in the cage trials. There might be several explanations for this difference. It is possible that foraging honey bees died before returning to the hive. Another possibility is that the bees used in the cage trials had limited access to the feed compared to the honey bees in the hives. The bees were not supplemented with pollen and honey other than sugar water in the cage trials due to *Nosema* spore contamination. Instead, a commercial dietary supplement was added to the 50% sucrose solution to compensate protein and vitamin needs of the honey bees. Honey bees have access to abundant pollen, honey and nectar stores in addition to the supplemental sucrose in the field. The fact that the honey bees in the hives have the opportunity to reach different feeds compared to those in the cage supported their survival for a longer period of time [62]. In addition, it has been reported that although a diet containing higher pollen content increased the density of *N*. *ceranae*, it prolonged the survival or lifespan of honey bees [67]. Also, honey bees fed with pollen have been showed to have a lower *Nosema* spore load than those fed with protein supplements [67].

## Conclusion

Significant findings were presented in this study on the high potential use of specific IgYs that do not leave residues in the honey and have less potential in terms of side effects compared to chemicals, in the treatment of *N*. *ceranae* infection in honey bees. In addition, specific IgYs were successfully obtained from egg yolk of chickens vaccinated with *N*. *ceranae* spores, for the first time in this study. It was determined that *Nosema*-specific IgY production in chickens vaccinated with four different methods showed fluctuations between the groups. Although the antibody titer remained high until the 7^th^ week following the last vaccination, it decreased gradually in the following weeks. The findings of this study showed that specific IgYs reduced the number of *Nosema*-infected honey bees and spore load significantly both in the field and in the cages under the controlled conditions. On the other hand, IgYs were detected to have no effect on honey bee mortality in the cage trials. Although a seven-day treatment protocol applied in the study revealed the effectiveness of IgY on *Nosema*, future studies with longer-term treatment protocols will provide more detailed information on this issue.

## Supporting information

S1 Raw images Raw images of blot/gel data shown Figs 1A, 1B, 2A and 2B. (PDF)
