# Supplementary material for: Production, characterization and therapeutic efficacy of egg yolk antibodies specific to Nosema ceranae
Source: PLoS One. 2024 Feb 9;19(2):e0297864. doi: 10.1371/journal.pone.0297864 (PMC10857605; doi:10.1371/journal.pone.0297864)

Lane 1 in the original figure below was used to generate Fig 1B.

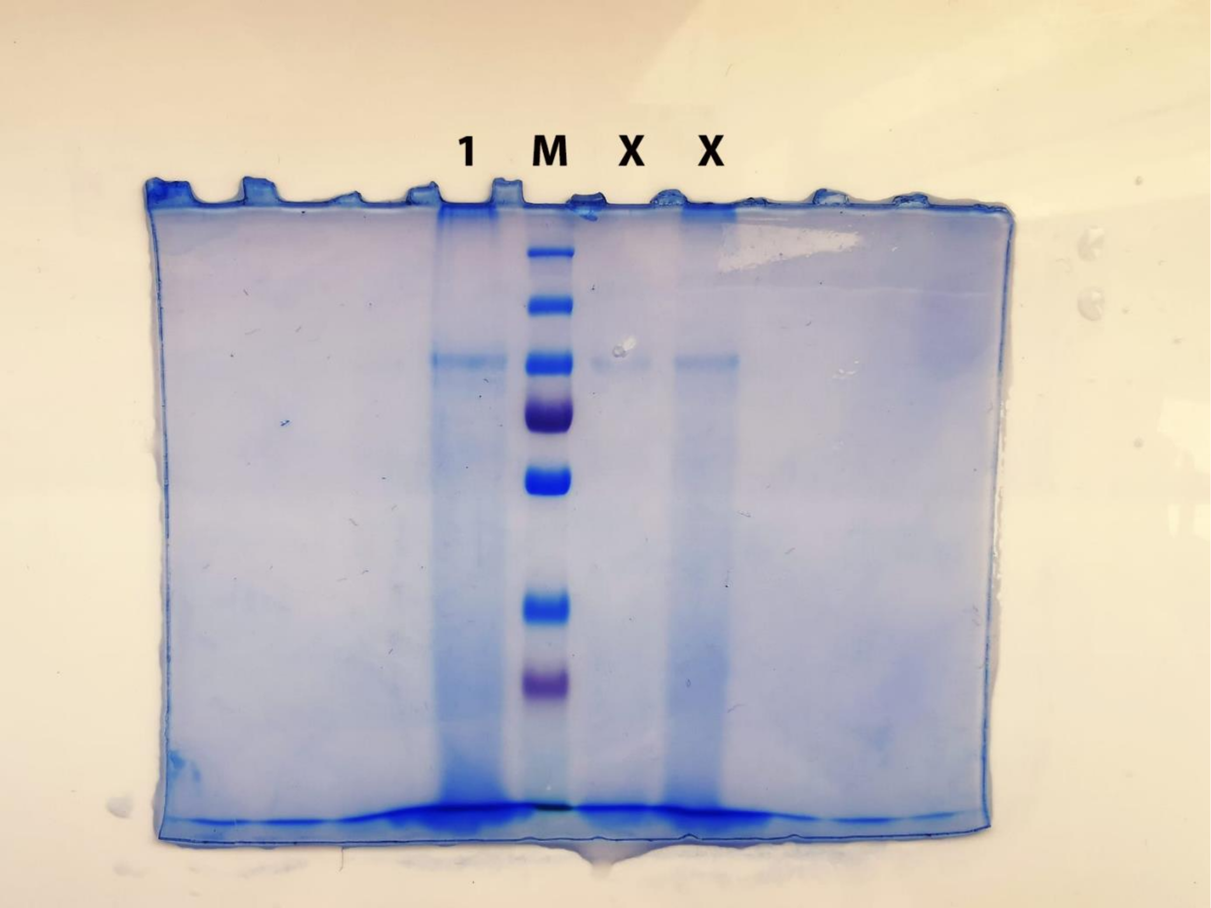

Lane 1 in the original figure below was used to generate Fig 1A.

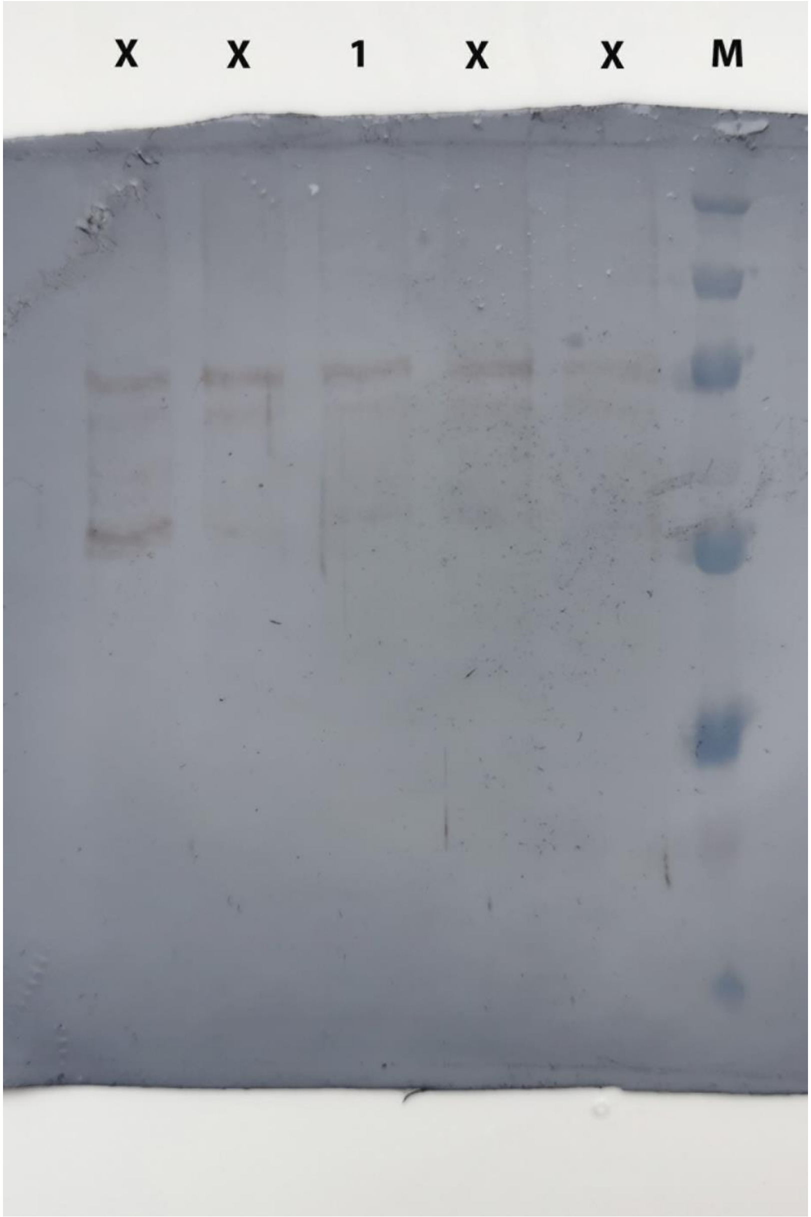

Lane 1 and 2 in the original figure below were used to generate Fig 2A.

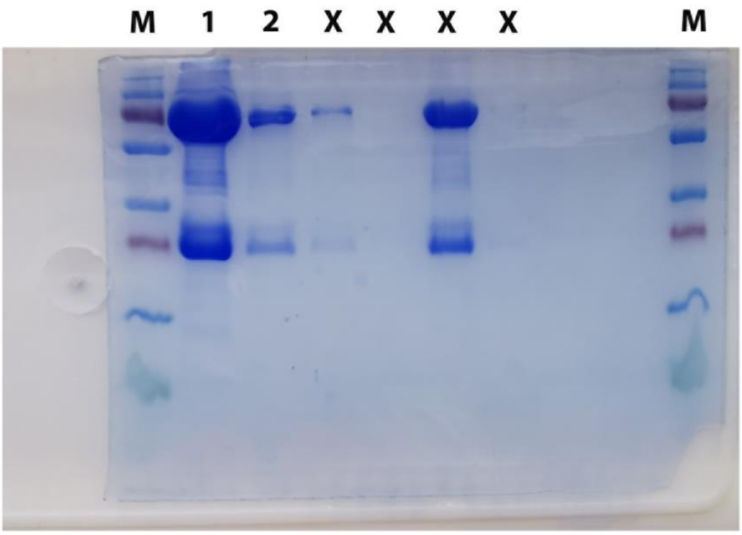

Lane 1 in the original figure below was used to generate Fig 2B.

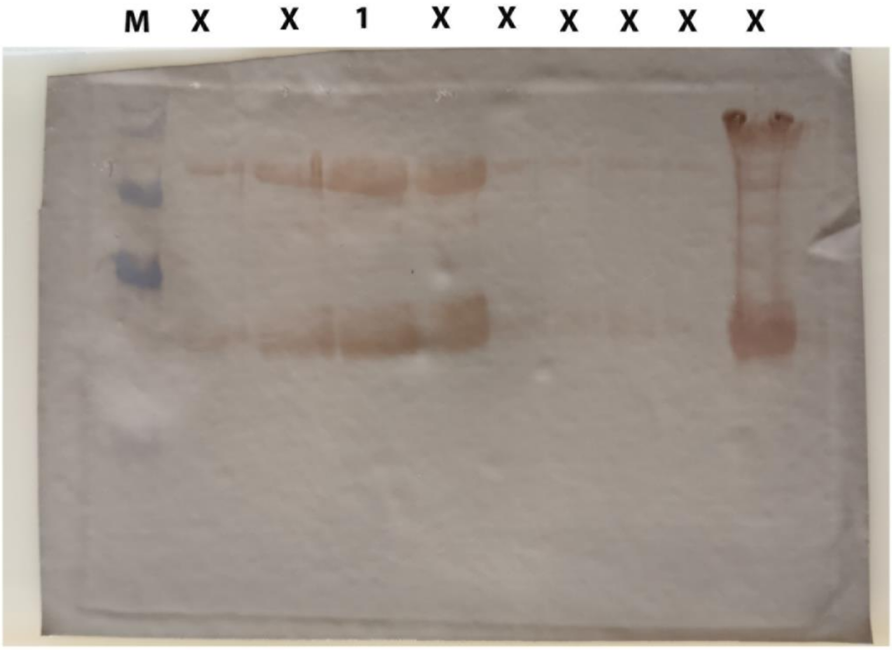

Supplement: S1 Raw images — (PDF) [file pone.0297864.s001.pdf]
